# Effects of Dipeptidyl-Peptidase 4 Inhibitor about Vascular Inflammation in a Metabolic Syndrome Model

**DOI:** 10.1371/journal.pone.0106563

**Published:** 2014-09-03

**Authors:** Nicolas F. Renna, Emiliano A. Diez, Roberto M. Miatello

**Affiliations:** 1 Department of Pathology, School of Medicine, National University of Cuyo, Mendoza, Argentina; 2 Laboratory of Cardiovascular Physiopathology, Institute of Experimental Medicine and Biology of Cuyo (IMBECU) - CONICET, Mendoza, Argentina; Bambino Gesu’ Children Hospital, Italy

## Abstract

**Background:**

In this study, we used vidagliptin(V) to examine the role of the DDP-IV, incretin system component, in the activation of different molecular inflammatory cytokines, NF-kB and VCAM-1 to generate a microenvironment that supports cardiovascular remodeling.

**Methods:**

Male WKY and SHR were separated into five groups: Control, FFR: WKY rats receiving a 10% (w/v) fructose solution during all 12 weeks, SHR, FFHR: SHR receiving a 10% (w/v) fructose solution during all 12 weeks and FFHR+V: (5 mg/kg per day for 6 weeks) (n = 8 each group). Metabolic variables and systolic blood pressure were measured. The TBRAS, eNOS activity, and NAD(P)H oxidase activity were estimated to evaluate oxidative stress. Cardiac and vascular remodeling were evaluated. To assess the cytokine, NF-kB and VCAM-1 immunostaining techniques were used.

**Results:**

The FFHR experimental model presents metabolic syndrome criteria, vascular and cardiac remodeling, vascular inflammation due to increased expression of NF-kB, VCAM-1, and pro-atherogenic cytokines. Chronic treatment with V was able to reverse total or partiality of variables studied.

**Conclusions:**

Data demonstrated an important effect of DDP-IV in reducing vascular inflammation, accompanied by a favorable reduction in metabolic and structural parameters.

## Introduction

Several authors recently reported that the incretin system induces an inflammatory and pro-lipolytic response via the PKA - NF-κB - IL-1 pathway and impairs insulin sensitivity and glucose uptake in human adipocytes [1]–[3]. One of the key mechanisms in the pathogenesis of diabetes-related vascular dysfunction is oxidative stress. Oxidative stress is attributable to excessive production of reactive oxygen species (ROS) and inflammatory markers by tumor necrosis factor-alpha (TNF-alpha), macrophage chemotactic protein-1 (MCP-1) and other markers [4]. The inflammatory response was reported to downregulate eNOS expression and upregulate iNOS expression in rodents and increase NAD(P)H oxidase activity and vascular remodeling [4]–[6].

Inhibitors of dipeptidyl peptidase-4 (DPP-IV), a key regulator of the actions of the incretin hormones, exert anti-hyperglycemic effects in type 2 diabetic patients. Long-term inhibition of DDP-IV may reduce pancreatic mass loss due to a reduction in inflammation induced by oxidative stress [7]–[8].

Spontaneously hypertensive rats (SHR) provide a model of genetic hypertension that allows the study of primary hypertension. The administration of carbohydrate-rich diets to rats can induce insulin resistance, hyperinsulinemia, dyslipidemia and moderate hypertension. Chronic fructose-fed rats (FFR) provide a useful experimental model for studying the interaction of the factors that shape metabolic syndrome [9]. We postulate that this dual experimental model could be appropriate for extrapolating results to human pathology [10].

In the present study, we used vidagliptin to examine the role of the DDP-IV, incretin system component, in the activation of different molecular inflammatory cytokines, NF-kB and VCAM-1 to generate a microenvironment that supports cardiovascular remodeling.

## Methods

### Animals and experimental design

All procedures were performed according to institutional guidelines for animal experimentation; the protocol was submitted and approved by the Institutional Committee for Laboratory Animal Use and Care (CICUAL) of the School of Medicine-UNCuyo. Thirty-day-old male Wistar Kyoto (WKY) and SHR rats were fed a standard commercial chow diet ad libitum and housed in a room under conditions of controlled temperature (20°C) and humidity with a 12-hour light/dark cycle during a 12-week experimental period. Vidagliptin (V) was administered to the respective groups during the last six weeks of the study.

I-Control (W): WKY rats receiving food and drinking water (DW) ad libitum;
II-SHR rats receiving food and DW ad libitum;
III-Fructose-fed Rats (FFR): WKY rats receiving a 10% (w/v) fructose (Parafarm, Buenos Aires, Argentina) solution in the DW during all 12 weeks,
IV-Fructose-fed Hypertensive Rats (FFHR): SHR rats receiving a 10% (w/v) fructose solution in the DW during all 12 weeks;
V-FFHR+V: FFHR receiving 5 mg/kg V by intraesophageal administration.

At the end of the experimental period, the rats were anesthetized with sodium pentobarbital (50 mg/Kg ip), blood samples were taken and the arteries and organs were aseptically excised for measurements.

### Systolic blood pressure measurement

The systolic blood pressure (SBP) was monitored indirectly in conscious, pre-warmed rats that were mildly restrained by the tail-cuff method and recorded on a Grass Model 7 polygraph (Grass Instruments Co., Quincy, MA, USA). The rats were trained in the apparatus several times before measurement.

### Biochemical Determinations

#### HOMA index and intra-peritoneal glucose tolerance test

The fasting plasma insulin was assayed using the ACS:180SE automated chemiluminescence system (Bayer, Germany). The plasma glucose levels were assayed using a commercial colorimetric method (Wiener Lab., Argentina). The homeostasis model assessment (HOMA) was used as an index to measure the degree of insulin resistance; it was calculated using the following formula: [insulin(µU/mL)×glucose(mmol/L)/22.5] [11].

Three days before the end of the experimental period, a glucose tolerance test (GTT) was performed. Rats fasted overnight were slightly anesthetized with pentobarbital, and glucose was administered (2 g/Kg ip). Blood samples were taken by tail-bleeding at 0, 30, 60 and 90 minutes after injection to determine the plasma glucose concentration. The total area under the curve was calculated as mmol/L/90 min.

#### Assessment of the lipid profile

At the end of the experimental period, blood samples were drawn from the animals after fasting for 12 hours. The total plasma cholesterol, HDL cholesterol and triglycerides were assessed using photocolorimetric enzymatic methods (Wiener Lab., Rosario, Argentina). The data are expressed in mmol/L.

### Oxidative Stress Determinations

#### Measurement of plasma thiobarbituric acid-reactive substances (TBARS)

To demonstrate the effect of increased oxidative stress at the vascular level, plasma lipid peroxidation was assessed by determining the TBARS concentration. This assay is based on the reaction between plasma malondialdehyde, a product of lipid peroxidation, and thiobarbituric acid, as has been previously described [12]. No correction for sample protein content was necessary because of the nature of sample [13].

### Measurement of vascular NAD(P)H-oxidase activity

The lucigenin-derived chemiluminescence assay was used to determine the NAD(P)H-oxidase activity in a segment of the thoracic aorta, as previously described [12]. To assess NAD(P)H-oxidase activity, NADPH (500 µmol/L) was added and chemiluminescence was immediately measured in a liquid scintillation counter (LKB Wallac Model 1219 Rack-Beta Scintillation Counter, Finland) set in the out-of-coincidence mode. Time-adjusted and normalized-to-tissue-weight scintillation counters were used for the calculations. The measurements were repeated in the absence and presence of diphenylene iodinium (DPI) (10–6 mol/L), which inhibits flavin-containing enzymes, including NAD(P)H oxidase [14]–[15].

### eNOS activity in homogenates of cardiac and arterial tissue

The activity of Ca^2+^/calmodulin-dependent endothelial nitric oxide synthase, (eNOS) was measured in mesenteric artery homogenates and in left-ventricle cardiac tissue based on the conversion of L-[3H]arginine into L-[3H]citruline. The values were corrected according to the protein contents in the homogenates (Bradford method) and the incubation time, and the results are expressed as dpm/mg protein/min. The material obtained from each animal was processed independently [16].

### Relative heart weight

To evaluate cardiac hypertrophy, we measured the relative heart weight (RHW). Briefly, the heart was separated from the great vessels, placed in a buffered saline solution (PBS), blotted with tissue paper to remove the blood, and weighed. The total heart weight was corrected according to the ratio between the heart weight (milligrams) and 100 grams of the total body weight before sacrifice.

### Tissue preservation

Tissue samples for histopathology were processed as has been previously reported (14). Samples from all rats were used for these observations. Anesthetized animals were briefly perfused with PBS (298 mOsmol/Kg H_2_O, pH 7.40, 4°C) to remove the blood. The mesenteric arteries were perfused in vivo with the same solution through the mesenteric artery for 5 min. For the histological studies, the arteries were also perfused with a 4% paraformaldehyde solution for 10 min and fixed in paraffin. Five-micron-thick tissue slices were transversely cut across the mesenteric tissue on a microstate (Microm HM, Germany) and processed for histological studies. A similar procedure was applied for heart tissue preservation by aortic retrograde perfusion.

### Quantitative Histomorphometry to determine cardiac hypertrophy

Histomorphological analyses were conducted on slices from the outer (free) wall of the left ventricle (LV) of the heart. Estimations of the cardiomyocyte area were made from sections stained with Masson trichrome solution. Areas with transverse sections of myofibers were selected. The contour of the fibers was then drawn manually. The total myocardiocyte area was expressed in square micrometers (µm2).

### Arterial structure

Changes in the structure of the arterial walls were assessed by measuring the medial layer of the mesenteric arteries. The slices were dyed and examined as has been previously described (14). Non-transverse sectioned arteries were excluded from the investigation. The lumen-to-media ratio (i.e., the ratio of the internal diameter to the medial thickness) (L/M) was then calculated. Fifty slices from each animal were processed and analyzed to obtain an average value for each rat. The average values were then used for the final analysis.

### SDS-PAGE and Immunoblot Analysis

The mesenteric tissue from each rat was washed in PBS, and the proteins were extracted in cold 20 mM Tris-HCl, pH 7.4, 150 mM NaCl, 10% glycerol, 1% Triton X-100, and a protease inhibitor mixture (P2714, Sigma). After sonication for 15 s (3 times with 10-s intervals) and extraction for 30 min at 4°C, the sample extracts were clarified by centrifugation at 14,000×g for 20 min and used immediately or stored at –20°C. The proteins were separated on 10% polyacrylamide slab gels and transferred to 0.22-µm nitrocellulose membranes (GE, Germany). Nonspecific reactivity was blocked by incubation for 1 h at room temperature in 5% non-fat dry milk dissolved in washing buffer (PBS, pH 7.6, 0.2% Tween 20). The blots were incubated with anti-p65 and anti-VCAM-1 antibodies (0.2 µg/mL in blocking solution) for 60 min at room temperature. Horseradish peroxidase-conjugated goat anti-rabbit-IgG and swine anti-goat-IgG dissolved in blocking buffer were used as secondary antibodies (0.25 µg/mL, 45 min at room temperature). Excess first and second antibodies were removed by washing 5 times for 5 min in blocking solution. Detection was accomplished with an enhanced chemiluminescence system (ABC, Dako System) and subsequent exposure to Kodak X-AR film (Eastman Kodak) for 5–30 s.

### Immunohistochemistry and digital confocal microscopy (IHC)

#### Detection of of transcription factors (WB)

A rabbit antibody against the C-terminus of rat NF-kB p65 subunit [Rel A] was obtained from Millipore International Inc. (Amsterdam, Netherlands) (AB1604b), and goat anti-rat VCAM-1 (C-19) antibody was obtained from Santa Cruz Biotechnology Inc. (Santa Cruz USA) (sc-1504). Tissue sections were cut at a 3-µm thickness from paraffin-embedded blocks. The antibodies were diluted 1∶1000. The primary incubations were carried out for 1 hour at 21–22°C, followed by extensive six washes in PBS with Triton X-100 for 5 min each. The secondary antibodies, anti-rabbit IgG TR and anti-goat IgG FITC (Sigma-Aldrich), were diluted in PBS alone according to the manufacturer’s instructions.

The images were collected with Nikon EZ-C1 3.00 software on a Nikon Diaphot TMD microscope.

### Measurement of the concentration of high-sensitive C Reactive Protein (hs-CRP)

The plasma HS-CRP concentrations were measured using a turbidimetric assay (Bayer Advia 1650, AG Leverkiusen). The data are expressed in mg/L.

### Cytokine determination by “ChemiArray”

The expression of the following cytokines was assessed using the ChemiArray system (rat antibody arrays) (Chemicon International, USA): neutrophil chemotactic cytokine 2 and 3 (CINC-2 and CINC-3), CX3CL1, monocyte chemotactic protein-1 (MCP-1), macrophage inflammatory protein-3 alpha (MIP-3 alpha), nerve growth factor beta (beta-NGF), tissue inhibitor of metalloproteinase-1 (TIMP-1), vascular endothelial growth factor (VEGF), granulocyte-macrophage colony-stimulating factor (GM-CSF), interferon gamma (INF-γ), interleukin 1 alpha and beta (IL-1α, IL-1β), interleukin 4, 6 and 10 (IL-4, IL-6, IL-10) LIX, leptin, tumor necrosis factor alpha (TNF-α). The procedures were carried out according to the manufacturer’s instructions. Detection was performed with a chemiluminescence system and subsequent exposure to Kodak X-AR film (Eastman Kodak) for 5–30 s. The cytokines were distributed in the membranes according the map (Table 1).

**Table 1:** Metabolic and cardiovascular variables.

Variable	WKY	FFR	SHR	FFHR	FFHR+V	
Fasting glucose (mmol/L)	4.88±0.1	6.44±0.2*	5.0±0.2	6.5±0.2*∧	4.6±0.1**	
Fasting triglycerides (mmol/L)	0.8±0.0	1.9±0.0* #	1.0±0.0	1.9±0.1* #	1.0±0.2**	
HOMA (µU/mLinsulin×mmol/L glucose)/22.5	4.00±0.2	11.0±0.1* #	7.1±0.15*	15.1±0.5* #∧	4.1±0.5**	
Area under glucose tolerancetest curve (mmol/L/90 min)	881±64	1352±21* #	1300±35*	1909±51* #∧	801±67**	
HDL Cholesterol (mg/dl)	21.5±0.3	13.2±0.4* #	19±1,0*	11.6±1,5* #∧	20.2±1.3**	
High-sensitivity C reactiveProtein (mg/dL)	1.5±0.1	3.5±0.0	3.1±0.1	4.5±0.1* #∧	1.01±0.3**	
Systolic bloodpressure (mmHg)						
Baseline	105±3	102±1.0	103±1	105±3	105±2	
6 weeks	110±1.0	129±2.0*	165±2*	172±3* #	170±3*	
12 weeks	112±1.3	139±3.0*	178±1* #	185±2* #∧	160±1.5**	

The above values correspond to metabolic and cardiovascular variables. Symbols indicate:

*p<0.001 vs. WKY;

∧p<0.001 vs. SHR;

# p<0.01 vs. FFR.

**vs. FFHR.

### Reagents

Unless otherwise noted, all reagents were purchased from Sigma Chemical Co, MO USA.

### Statistical and Data Analysis

The data are expressed as the mean ± SEM. The statistical significance of the comparisons between all groups was assessed by one-way ANOVA followed by the Bonferroni post-test. A two-sided p value of less than 0.05 was considered significant.

## Results

### Long-Term DPP-4 Inhibition Improves Metabolic Indexes and Blood Pressure

Chronic administration of fructose induced several alterations included in the cluster of risk factors that characterizes MS. The comparison between the HOMA index and the areas under the GTT curve indicated that the FFR and FFHR developed glucose intolerance, as demonstrated by the significantly increased HOMA index and area values compared to the control rats (Table 1).

On the other hand, the animals in the FFR and FFHR groups also showed significant differences in the levels of triglycerides and HDL-cholesterol when compared to the controls (Table 1). The SHR, FFR and FFHR groups also showed significant differences in the levels of hs-CRP when compared to the WKY group. The FFHR group showed higher hs-CRP levels than the other groups. V reduced all these variables to the levels of WKY (Table 1).

Table 1 also shows the time-course of the changes in SBP during the experimental period. By the sixth week, the SBP values of the FFHR and SHR groups were significantly increased compared to the control group, and there was an increase in pressure in the FFR group, which was lower but still significant. Treatment with V did not return the SBP values to those of the control animals. These results on the value of blood pressure are statistically significant but not clinically significant, because the reduction of this value was not less than 140 mmHg.

### Long-Term DPP-4 Inhibition results in the recovery of the oxidative status

Table 2 shows that NAD(P)H-oxidase activity and the plasma TBARS concentration was significantly higher in the aortas of FFHR when compared to those from other groups. In addition, in the FFHR group, the arterial eNOS activity was significantly reduced, contributing to a decrease in the production and consequent bioavailability of nitric oxide (NO).

**Table 2:** Oxidative stress and morphometric variables.

Variable	WKY	FFR	SHR	FFHR	FFHR+V
NAD(P)H oxidase activity(counts/min/mg tissue)	40.5±6	133±5*	160±9.1* #	297±9.1* #∧	42±9.1*∧**
Arterial eNOS activity(dpm/mg/prot/min)	85.0±2	60.1±2.6*	80.0±2.1	56.4±5.7* #∧	86.4±1.1**
TBARS (µmol/L)	1±0.1	2.2±0.1*	1.69±0.1*	2.8±0.1* #∧	1.03±0.6#∧**
Relative heart weight(mg/100 g body weight)	225±4	290±4*	330±1.8* #	400±4* #∧	302±6**
Myocardiocyte area (µm^2^)	1682±69	2066±57*	2222±78* #	3242±55* #∧	1788±34**
Lumen/media relationshipmesenteric arteries	13.9±0.3	10.2±0.5*	8.9±0.6* #	8.45±0.2* #	13.8±4**

The above values correspond to oxidative stress and morphometric variables. Symbols indicate:

*p<0.001 vs WKY;

∧p<0.001 vs SHR;

# p<0.01 vs FFR.

**vs FFHR.

Administration of V effectively reduced superoxide production by reducing the activity of NAD(P)H oxidase and TBARS, and it was also able to normalize the eNOS activity (Table 2). Furthermore, most likely by inhibiting the activity of NAD(P)H oxidase, V was also able to achieve these effects, normalizing the endothelial oxidative status (Table 2).

### Long-Term DPP-4 Inhibition modifies cardiac and vascular hypertrophy

The RHW and myocardiocyte area was significantly higher in the FFR, SHR and FFHR groups than in the control rats, demonstrating myocardial hypertrophy in these experimental models (Table 2). The FFHR group always displayed a significantly reduced L/M when compared to the corresponding arteries from the WKY group; this result was also observed in the FFR and SHR groups. Chronic treatment with V partiality reduced myocardial hypertrophy and vascular remodeling in the FFHR model. Table 2.

### Effects of DDP-IVi on vascular inflammation

**Figure 1:**
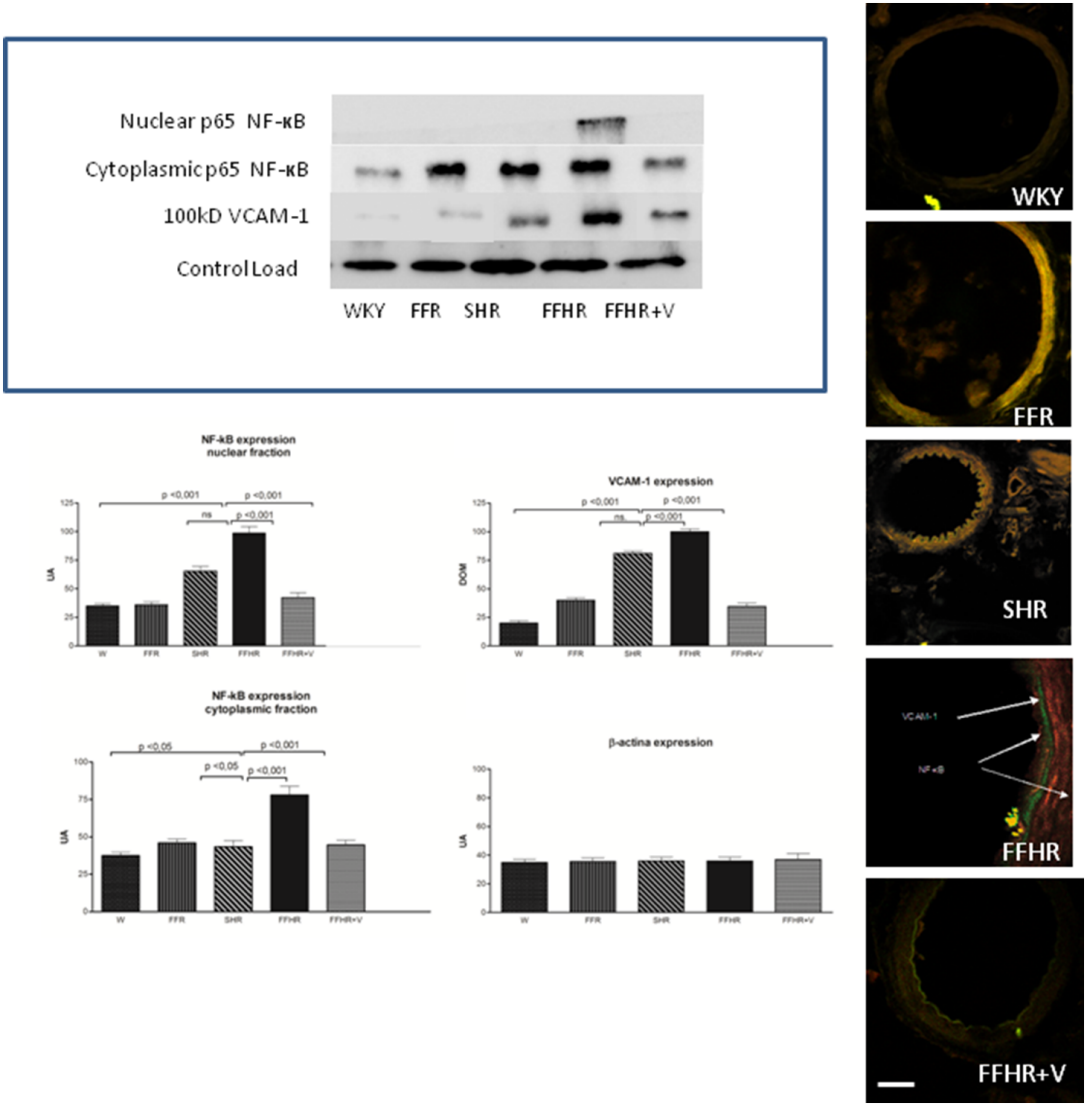
Cytoplasmatic and nuclear p-65 fraction of NF-κB and VCAM-1 expression in mesenteric arteries detected using WB and IHC. The upper panel shows a representative WB probed with anti-VCAM-1-FITC and anti-p65-TRITC. The results represent the optical density of the bands for each group. The lower panel shows microphotographs of mesenteric tissue obtained using a laser ICM 600x.

The expression of NF-kB and VCAM-1, two products that actively participate in vascular inflammation, is shown in Figure 1. Both molecules were detected by IHC. The expression of these molecules in the FFHR group increased significantly compared to the control group (W). The right panel shows a representative image of a WB for these proteins. The average optical density significantly increased in mesenteric artery homogenates from the FFHR and FFR groups compared to the controls. It can be observed that the distribution of the control load was similar for all groups.

In IHC images of the vascular wall, the FFHR group shows a large increase in NF-kB expression at the level of the EC and VSMC, and VCAM-1 is expressed at the sub-endothelial level. V reduced the activation and nuclear translocation of NF-κB (p65 fraction) and VCAM-1 expression. This could be explained because in the FFHR group, nuclear factor NF-κB production was more important due to the production of superoxides as result of the insulin-resistance status. This may also be a determining factor in the reduction of vascular remodeling, as previously shown.

### DDP-IV on activation of local cytokines

**Figure 2:**
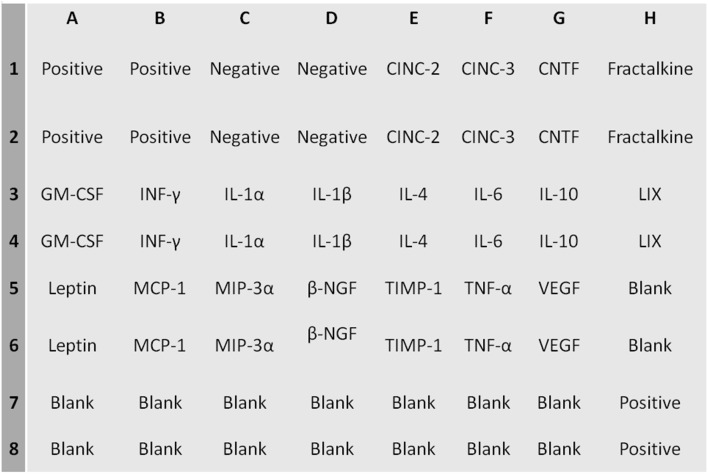
ChemiArray Rat Lysate Cytokine Antibody Array I Map.

**Figure 3:**
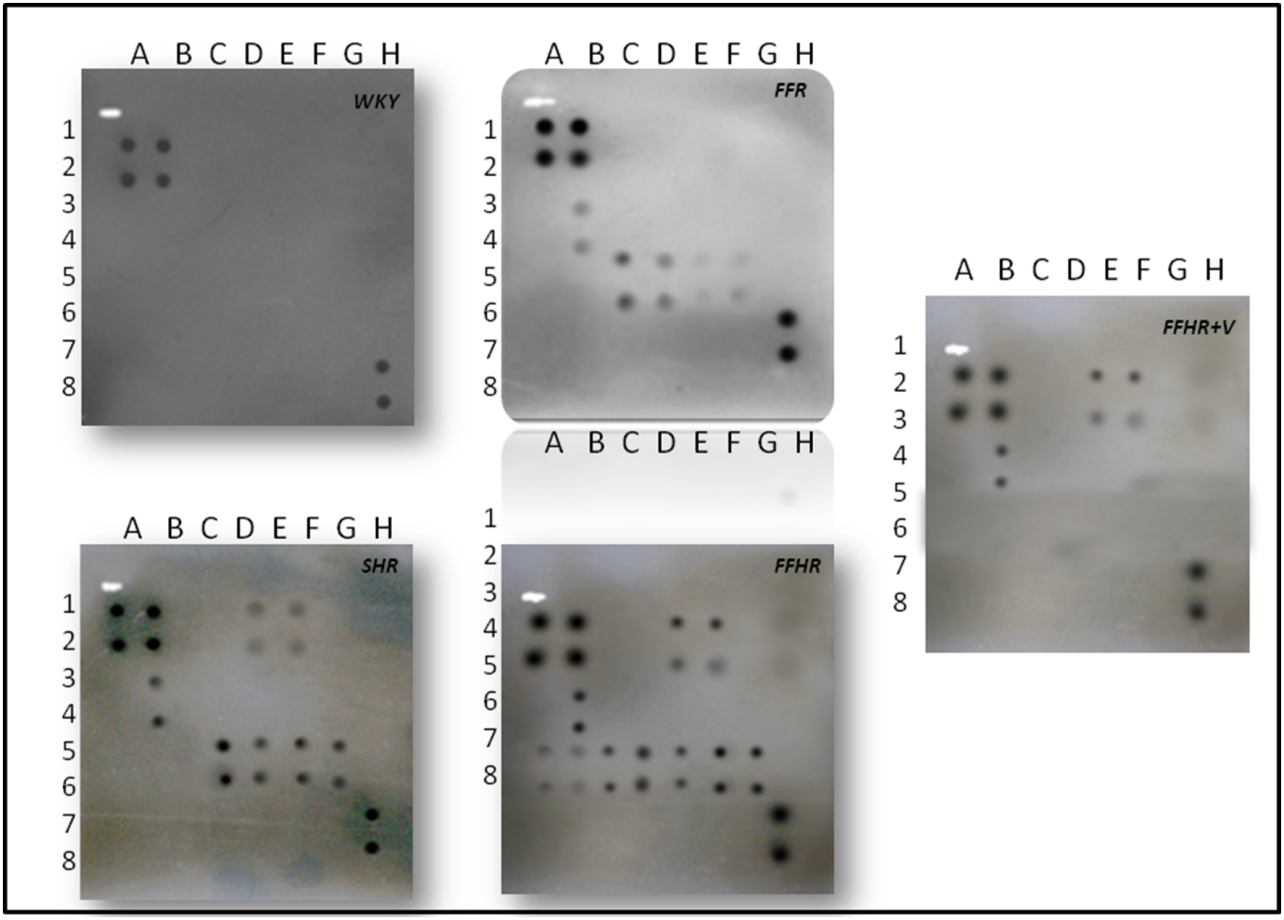
Detection of cytokines on membrane antibody arrays by chemiluminescence. Duplicate spots in the following locations represent each cytokine. See Figure 1. The average net light intensity for each pair of cytokine spots detected based on ray-scale levels using US NIH Image software ver 1.66. Cytokines names: Neutrophil chemotactic cytokine 2 and 3 (CINC-2 and CINC-3), ciliary neurotrophic factor (CNFT), monocyte chemotactic protein-1 (MCP-1), inflammatory protein macrophage-3 alpha (MIP-3 alpha), nerve growth factor beta (beta-NGF), tissue inhibitor of metalloproteinase-1 (TIMP-1) and vascular endothelial growth factor (VEGF), granulocyte colony stimulating factor, macrophage (GM-CSF), interferon gamma (INF-γ), interleukin 1 alpha and beta (IL-1α, IL-1β), interleukin 4, 6 and 10 (IL-4, IL-6, IL-10), lipopolysaccharide induced CXC chemokine (LIX or CXCL5), leptin, tumor necrosis factor alpha (TNF-α).

In FFHR model, we observed a significant increase in the levels of several cytokines, including the following: CINC2, CINC3, VEGF, MIP-3, beta-NGF, VEGF, Leptin, TNF-alpha, INF-gamma and MCP-1 (Figures 2, 3 and Table 3). This finding is the first evidence of the presence of local inflammation at the vascular level in an experimental model of insulin resistance, such as FFHR. This result shows the significant presence of pro-atherogenic cytokines, such as CINC2, CINC3, VEGF, Leptin, TNF-alpha, MCP-1 and TIMP-1, as well as others whose role at the vascular level is unknown, such as MIP-3 and beta-NGF, INF-gamma and others with anti-atherogenic effects. Table 3.

**Table 3:** Cytokine release profiles on different experimental models.

Cytokine^a^	Relative levels^b^	Array Location^c^	Fold increase for control group (WKY)^d^			
			SHR	FFR	FFHR	FFHR+V
CINC-2	H	E1-2	1.18	1.10	2.42	2.35
CINC-3	H	F1-2	1.34	1.30	2.74	2.67
CNTF	-	G1-2	∼	∼	∼	∼
Fractalkine	-	H1-2	∼	∼	∼	∼
GM-CSF	-	A4-5	∼	∼	∼	∼
INF-γ	H	B3-4	5.00	4.50	5.90	5.40
IL-1α	-	C3-4	∼	∼	∼	∼
IL-1β	-	D3-4	∼	∼	∼	∼
IL-4	-	E3-4	∼	∼	∼	∼
IL-6	-	F3-4	∼	∼	∼	∼
IL-10	-	G3-4	∼	∼	∼	∼
LIX	-	H3-4	∼	∼	∼	∼
Leptin	H	A5-6	1.16	1.33	2.10	1.07
MCP-1	H	B5-6	1.25	NC	4.00	1.02
MIP-3α	H	C5-6	1.16	1.33	3.91	3.80
β-NGF	H	D5-6	2.40	2.40	3.50	3.53
TIMP-1	H	E5-6	2.60	1.52	2.50	∼
TNF-α	H	F5-6	3.19	1.10	3.35	∼
VEGF	H	G5-6	2.50	NC	3.05	1.05

a Name of cytokine.

b Relative levels: -, undetectable; H, high: L, low.

c See Figure 3 for the location of the duplicate spots in the matrix.

d When the control “∼” symbol was used to indicate an approximation of zero, the values indicate the fold increase vs. WKY (control group). NC, no change (less than or equal to the fold change in the WKY group).

Chronic treatment with V returned the expression levels of pro-atherogenic cytokines, including VEGF, Leptin, TIMP-1 and MCP-1, to control values, and V administration significantly reduced MIP-3alpha and TNF-alpha, demonstrating the important role that the incretin system plays in vascular inflammation in this experimental model (Table 3).

## Discussion

In this article, we demonstrated an important effect of DDP-IV in reducing vascular inflammation, accompanied by a favorable reduction in metabolic and structural parameters.

The FFHR experimental model presents hypertension, dyslipidemia, insulin resistance, vascular and cardiac remodeling, inflammation demonstrated by increased hsCRP and vascular inflammation due to increased expression of NF-kB, VCAM-1 and pro-atherogenic cytokines. The increased expression of VCAM-1 is a marker of vascular inflammation, vascular permeability and endothelial dysfunction [17]–[19].

The inflammatory process identified in this experimental model has two components: 1- a local component involving an increase in the levels of nuclear transcription factors with subsequent activation of the inflammatory cascade, resulting in a strong presence of cytokines, and level 2– a systemic component involving increased hepatic synthesis of CRP due to a probable increase of IL-6 [20]–[22].

The data suggest that incretin system dysfunction, as happen in patients with diabetes mellitus or metabolic syndrome, allows activation of inflammatory response in different levels. With the consequent creation of a vascular microenvironment that is conducive to the creation, perpetuation, progression, and destabilization of vascular injury, with either a simple eutrophic mechanism of vascular remodeling, or the generation of an atherosclerotic lesion.

A number of mechanisms may underlie these results. Given that GLP-1 is a physiological substrate of DPP-IV, DPP-IV inhibition by V may be expected to increase the circulating levels of GLP-1 [23] Several studies have reported beneficial effects of GLP-1 on the cardiovascular system. In humans, Nikolaidis et al. [24] have shown that a 72-h infusion of GLP-1 improved left ventricular function in patients with acute myocardial infarction and systolic dysfunction after successful reperfusion therapy, an effect that was observed in both diabetic and nondiabetic patients. The authors suggested that this observation might be explained by the insulinotropic and insulinomimetic properties of GLP-1; alternatively, GLP-1 might also improve endothelial function. Studies have shown that GLP-1 improves endothelium-dependent vascular responses in the brachial artery while leaving endothelium-independent responses unaffected in healthy humans and patients with type 2 diabetes [25]–[26]. The cardiovascular actions of GLP-1 may occur either directly through the GLP-1 receptor or through a GLP-1 receptor-independent effect of the degradation product of GLP-1, GLP-1(9–36) [27].

In addition to GLP-1, DPP-IV also degrades GIP, and potentially cytokines and certain chemokines (including stromal-derived factor 1-α). Thus, other substrates of DPP-IV may be responsible for the improvement in endothelial function. Alternatively, V might improve endothelial function by influencing insulin and glucose levels. Insulin causes vasodilatation by increasing endothelial production of NO [28]. Vildagliptin might have a direct pharmacological, yet unidentified, effect on the endothelium.

The improvement in endothelial function and oxidative stress could result in a decrease in activation of the inflammatory process.

Other authors have suggested that the DDP-IV inhibitors may have anti-inflammatory effects, such as reduced activation of TNF-alpha during macrophage activation [29].

Our results suggest previously unidentified broad pleiotropic effects [30] of DDP-IV inhibitors and indicate a potential role of vascular inflammation modulators, which may allow for the reduction of the vascular complications of atherosclerosis related of metabolic syndrome.
